# Cigarette smoke toxin hydroquinone and misfolding pancreatic lipase variant cooperatively promote endoplasmic reticulum stress and cell death

**DOI:** 10.1371/journal.pone.0269936

**Published:** 2022-06-15

**Authors:** Norbert Kassay, Vanda Toldi, József Tőzsér, András Szabó

**Affiliations:** 1 Department of Biochemistry and Molecular Biology, Faculty of Medicine, University of Debrecen, Debrecen, Hungary; 2 Doctoral School of Molecular, Cell and Immune Biology, University of Debrecen, Debrecen, Hungary; University of Lille, FRANCE

## Abstract

Mutation-induced protein misfolding of pancreatic secretory enzymes and consequent endoplasmic reticulum stress can cause chronic pancreatitis. A recent study revealed that cigarette smoke also increases the risk of the disease through endoplasmic reticulum stress. Here, we investigated the cumulative cellular effect of the G233E misfolding human pancreatic lipase variant and hydroquinone; a main toxic constituent of cigarette smoke, using mammalian cell lines. We found that hydroquinone reduces cell viability on a dose-dependent manner through programmed cell death, and diminishes lipase secretion without affecting its expression. Interestingly, hydroquinone decreased the viability more markedly in cells expressing the G233E lipase variant, than in cells producing wild-type lipase. The more substantial viability loss was due to increased endoplasmic reticulum stress, as demonstrated by elevated levels of X-box binding protein 1 mRNA splicing and immunoglobulin binding protein, NAD(P)H:quinone oxidoreductase 1 and C/EBP homologous protein expression. Unresolved endoplasmic reticulum stress, and especially up-regulation of the pro-apoptotic transcription factor C/EBP homologous protein were likely responsible for the increased cell death. Our observations demonstrated that the combination of hydroquinone and misfolding pancreatic lipase variant promote increased levels of endoplasmic reticulum stress and cell death, which may predispose to chronic pancreatitis.

## Introduction

Chronic pancreatitis is a progressive inflammatory disorder of the human pancreas, frequently caused by environmental factors such as alcohol and cigarette smoke. Several clinical studies reported dose dependent correlations between smoking and chronic pancreatitis [1–3]. A recent publication demonstrated that cigarette smoke together with alcohol consumption contribute to the pathogenesis of chronic pancreatitis through endoplasmic reticulum (ER) stress in pancreatic acinar cells [4]. Acrolein, crotonaldehyde and hydroquinone (HQ) are the most cytotoxic chemicals of the thousands of cigarette smoke constituents [5]. Several publications described that these toxins cause cell death via the activation of ER stress pathways [4, 6–8]. In addition to environmental risk factors, genetic predisposition also contributes to the pathogenesis of chronic pancreatitis. Mutations in pancreatic secretory enzymes such as cationic trypsinogen (PRSS1), procarboxypeptidase A1 (CPA1), carboxyl ester lipase [3] and pancreatic lipase (PNLIP) may lead to protein misfolding in pancreatic acinar cells [9]. Due to rigorous quality control in the ER, highly expressed misfolded proteins may retain in the cells, eliciting ER stress and consequent acinar cell injury.

During ER stress, three sensor proteins (IRE1α, PERK and ATF6) located in the ER membrane may be activated, and trigger the so-called unfolded protein response [10, 11]. Binding of the ER chaperone and master regulator immunoglobulin binding protein (BiP) to the ER sensors interfere with their function. Increased levels of misfolded proteins draw BiP away from the sensors, by which those become activated and initiate downstream signal transduction pathways. X-box binding protein 1 (XBP1) is a transcription factor of the unfolded protein response. IRE1α acts as an endoribonuclease that excites a short intron from XBP1 mRNA, allowing XBP1 protein to be translated. Unresolved ER stress may stimulate the expression of the pro-apoptotic transcription factor C/EBP homologous protein (CHOP) through PERK and ATF6 pathways. The activation of PERK pathway may also result in the expression of the NAD(P)H:quinone oxidoreductase 1 protein (NQO1) responsible for cellular adaptation to stress [12].

PNLIP is one of the major hydrolase enzymes in the human gastrointestinal system responsible for dietary triacylglycerol digestion. Recently, a rare homozygous PNLIP mutation (T221M) associated with lipase deficiency was identified in two members of an Arab family in Israel [13]. The mutation also resulted in exocrine insufficiency, suggesting that the patients had pancreatitis. Functional characterization revealed that the T221M mutation caused ER stress through lipase misfolding and accumulation as intracellular protein aggregates [14]. More recently, four additional uncommon heterozygous PNLIP mutations (A174P, G233E, C254R and V454F) characterized by defective lipase secretion and incomplete penetrance were described in European chronic pancreatitis cohorts [15]. The mutations induced intracellular PNLIP misfolding and ER stress, which increased susceptibility to chronic pancreatitis [16]. Nevertheless, based on the variable clinical data, these heterozygous mutations alone are unlikely to cause the disease. Thus, concomitant exposure to other risk factors is required for the development of chronic pancreatitis.

In the present study, we used the common human embryonic kidney (HEK) 293AD cells characterized by improved cell adherence, and the pancreatic AR42J cells that are well suited for research on pancreatitis to compare the cytotoxicity of cigarette smoke toxins acrolein, crotonaldehyde and HQ. Given the fact that both genetic predisposition, and smoking can cause chronic pancreatitis via ER stress, we used the G233E misfolding PNLIP variant as an example, and examined if the detrimental effects of secretory protein misfolding and HQ cooperatively enhance the susceptibility to the disease.

## Materials and methods

### Expression plasmids and adenovirus vectors

Construction of wild-type and G233E PNLIP-10His-pcDNA3.1(-) expression plasmids and PNLIP-10His adenoviral vectors were reported previously [14–16]. Adenoviruses were amplified using HEK 293AD cells (Agilent, #240085) and liberated from the cells by repeated freeze-thaw cycles [16]. Adenoviral vectors were purified with Pierce Strong Anion Exchange Mini Spin Columns (Thermo Scientific) based on the instructions of the AdenoONE purification kit (Sirion Biotech). The columns were washed with 0.4 mL 0.1 M NaOH, centrifuged for 1 min at 2,000 g, and washed again with 0.4 mL 0.1 M sodium acetate buffer (pH 5.0) and centrifuged. The columns were equilibrated twice with 0.4 mL purification buffer (50 mM HEPES, 2 mM MgCl2, 0.1% Tween-20, pH 8.0) each time followed by centrifugation. Cell lysates containing adenoviruses were diluted with ten-volumes of purification buffer, loaded onto the columns and centrifuged for 3 min at 2,000 g. The columns were washed with 0.4 mL purification buffer supplemented with 0.2 M NaCl, and adenoviruses were eluted with 0.4 mL purification buffer supplemented with 1 M NaCl. Purified adenoviruses were mixed with 0.1 mL 50% glycerol and stored in aliquots at -70°C. Typical titers of PNLIP adenoviruses were 109–1010 IFU/mL determined by the AdEasy Viral Titer Kit (Agilent). Adenoviral stocks were stable over 10 months of storage.

### Cell cultures and gene delivery

HEK 293AD were maintained in DMEM cell culture medium (high glucose DMEM supplemented with 10% FBS, 4 mM glutamine, 50 U/ml penicillin and 50 μg/ml streptomycin) at 37°C in a standard cell culture incubator. One day prior to experiments, cells (180,000/well) were cultured in a 12-well tissue culture plate in 0.5 mL culture media. The preparation of branched polyethylenimine stock was described previously [17]. Transfections were carried out with 2 μg plasmid DNA in 0.1 mL Opti-MEM (Gibco) media containing 12 μL polyethylenimine stock. After 6 h incubation, the media were removed, the wells were rinsed with phosphate buffered saline (PBS) and 1 mL Opti-MEM supplemented with 50 U/ml penicillin and 50 μg/ml streptomycin was added into each well. AR42J rat pancreatic cells (CLS Cell Lines Service, #500478) were maintained as described previously [18]. One day prior to experiments, the cells were cultured in a 12-well plate (400,000/well) and incubated at 37°C in the presence of 100 nM dexamethasone. After 48 h incubation, the cells were rinsed with PBS and transduced with adenoviral vectors (final 5 x 107 IFU/mL) in 1 mL Opti-MEM supplemented with 50 U/ml penicillin, 50 μg/ml streptomycin and 100 nM dexamethasone.

### Chemical treatments

Acrolein, crotonaldehyde and hydroquinone (HQ) were obtained from Sigma-Aldrich. Prior to the experiments, stock solutions of acrolein and crotonaldehyde (200 mM) were freshly prepared using DMSO as a solvent. HQ powder was dissolved in water (200 mM) and stored in aliquots at -20°C until use. HEK 293AD and AR42J cells were incubated with 10–200 μM final concentrations of the chemical reagents as indicated in Figure legends.

### MTT assay

MTT reagent (3-(4,5-dimethylthiazol-2-yl)-2,5-diphenyltetrazolium bromide, Invitrogen) was dissolved in PBS at 5 mg/mL concentration. In each well of a 12-well-plate, conditioned media were replaced with 1 mL fresh Opti-MEM supplemented with 0.1 mL MTT reagent. Formazan crystals were developed after 10–60 min incubation at 37°C. The staining solution was removed from the wells and 0.2 mL DMSO was pipetted into each well and mixed thoroughly to dissolve the dark blue crystals. The dissolved formazan was transferred into a well of a 96-well-plate and its absorbance was determined with a plate reader at 544 nm.

### Flow cytometry

Media from HEK 293AD and AR42J cells cultured in 12-well plates were removed and the cells were harvested by the addition of 1 mL 0.06% trypsin (Gibco) into each well. Cells were transferred into microtubes, washed with 1 ml cold PBS and centrifuged at 100 g for 5 min. Cells were stained with FITC annexin V and propidium iodide (PI) using the FITC Annexin V / Dead Cell Apoptosis Kit (Invitrogen) following the manufacturer’s protocol. Stained cells were filtered with MES filters (41 μm), transferred into FACS tubes and analyzed by BD FACSAria III flow cytometer (BD Biosciences) using 488 nm laser with 530/30 BP filter for FITC and 561 nm laser with 610/20 BP filter for PI.

### SDS-polyacrylamide gel electrophoresis and immunoblotting

Proteins in the conditioned media of HEK 293AD (200 μL) and AR42J (40 μL) were precipitated with 10% trichloroacetic acid 48 h after transfection and centrifuged for 10 min at 17,000 g. Pellets were reconstituted with Laemmli buffer supplemented with 100 mM dithiothreitol and heat denatured at 95°C for 5 min. Proteins were resolved with 12% SDS-polyacrylamide gel electrophoresis followed by staining with Coomassie Brilliant Blue R-250. HEK 293AD and AR42J were lysed as reported earlier [16]. PNLIP and GAPDH in total protein cell lysates were analyzed with 12% SDS-PAGE and immunoblotting as described previously [16].

### RT-PCR and qPCR analyses

Total RNA was isolated from cells by NucleoSpin RNA Plus kit (Macherey-Nagel), from which 2 μg was reverse transcribed by High Capacity cDNA Reverse Transcription Kit (Applied Biosystems). The extent of XBP1 mRNA splicing in HEK 293AD and AR42J cells was determined by PCR using human and rat XBP1 primer pairs [14, 19]. PCR products were resolved on 2% agarose gel, stained with GelRed (Biotium) and evaluated by densitometry using the Quantity One program (BioRad). Gene expression levels of BiP, CHOP, NQO1 and GAPDH in the cDNA samples were determined by qPCR as described recently [16] using Thermo Scientific human or rat BIP/HSPA5 (Hs00607129_gH, Rn00565250_m1), CHOP/DDIT3 (Hs00358796_g1, Rn00492098_g1), NQO1 (Hs00168547_m1, Rn00566528_m1) and GAPDH (Hs02758991_g1, Rn01775763_g1) TaqMan probes.

### Statistical analysis

Data are presented as mean ± SD. Statistical analyses were performed with two-way analysis of variance (ANOVA) technique using GraphPad 8.0.1 (Prism Inc). P values <0.05 were considered significant.

## Results

### Effect of major cigarette smoke components on the viability of HEK 293AD cells

**Fig 1:**
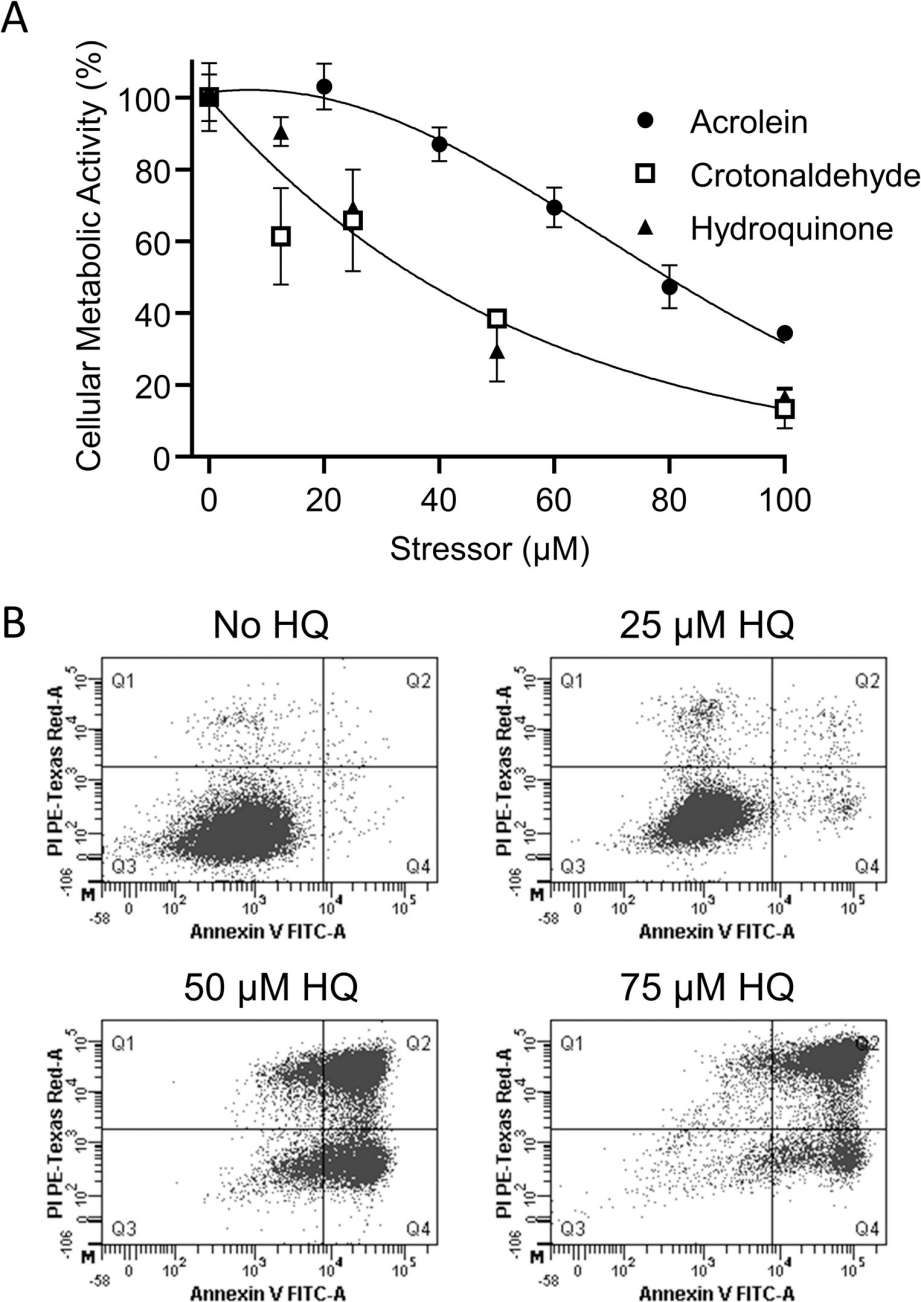
Viability of HEK 293AD cells in the presence of main smoke constituents. A) Cells were treated with indicated concentrations of acrolein, crotonaldehyde or hydroquinone (HQ). After 24 h incubation at 37 ˚C the levels of cellular metabolic activity were determined by MTT assay as described in Materials and Methods. Values are expressed as percent of untreated cells. Data points represent the average of two or three independent experiments with SD. B) Cells were exposed to indicated concentrations of HQ. After 24 h incubation at 37 ˚C, cells were stained with FITC annexin V and propidium iodide, and analyzed by flow cytometry as described in Materials and Methods. Fluorescence of FITC annexin V and propidium iodide are shown on the x and y axes, respectively. Representative pictures of three experiments are shown.

In order to characterize the detrimental effect of common cigarette smoke constituents on HEK 293AD, we incubated the cells in the absence or presence of increasing concentrations of acrolein, crotonaldehyde and HQ, and inferred cell viability by measuring cellular metabolic activity (Fig 1A). Acrolein up to 20 μM did not influence cell metabolism, whereas at higher concentrations, a gradual decrease in metabolic activity was observed. In contrast, crotonaldehyde and HQ, even at low concentrations, progressively reduced cell viability, as more than 80% loss of cellular metabolic activity was observed in the presence of 100 μM crotonaldehyde and HQ. Due to its significant impact on metabolic activity and high storage stability, the effect of HQ was further investigated in detail. In order to study whether decreased cellular metabolism of HEK 293AD is associated with programmed cell death or necrosis, the amount of FITC annexin V and propidium iodide stained cells was monitored in the absence or presence of increasing concentration of HQ with flow cytometry (Fig 1B). HQ up to 25 μM did not decrease cell viability, whereas in the presence of 50 and 75 μM HQ, programmed cell death occurred first, indicated by strong staining with FITC annexin V followed by increased staining with propidium iodide.

### Effect of HQ on PNLIP secretion in HEK 293AD

**Fig 2:**
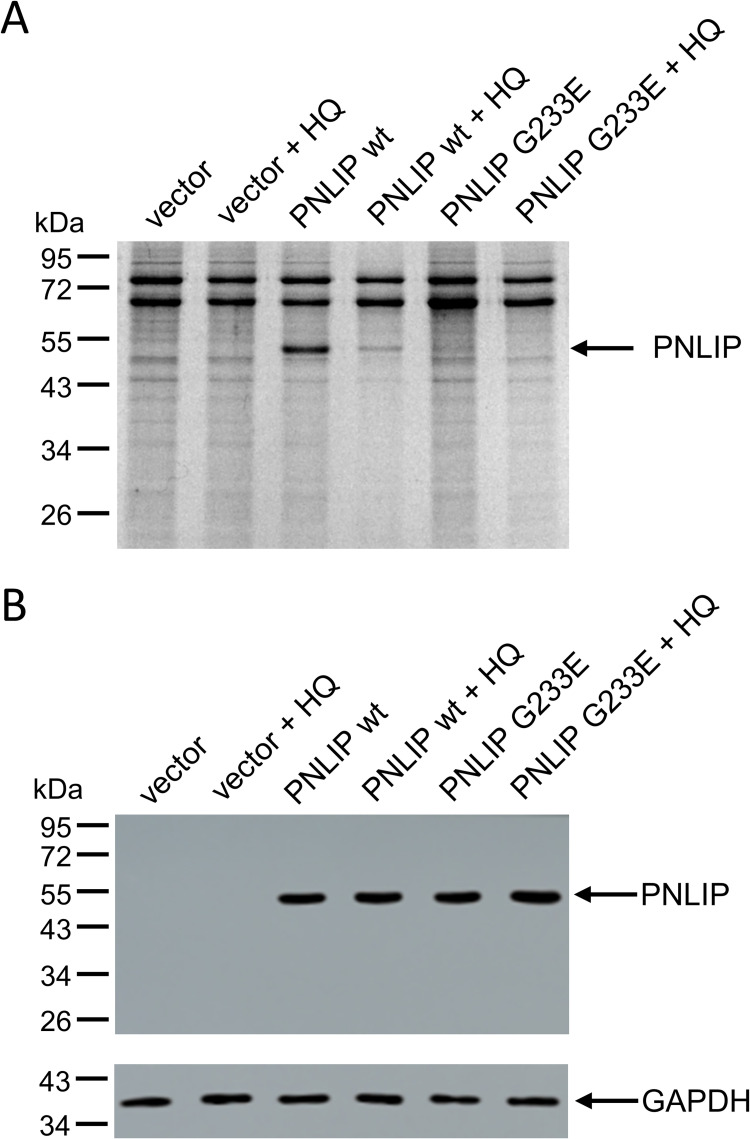
Effect of hydroquinone (HQ) on PNLIP secretion in HEK 293AD cells. One day post-transfection, cells were incubated in the absence or presence of 25 μM HQ for 24 h at 37 ˚C. A) PNLIP protein in the conditioned media was analyzed by SDS-PAGE and Coomassie staining as described in Materials and Methods. B) PNLIP and GAPDH proteins in cell lysates were monitored by SDS-PAGE and immunoblotting as described previously [16].

Previous studies demonstrated that G233E mutant PNLIP is expressed but not secreted by transfected HEK 293T cells due to protein misfolding, whereas the wild-type PNLIP was readily expressed and secreted [15, 16]. In the present study, we incubated HEK 293AD cells expressing wild-type and G233E mutant PNLIP with 25 μM HQ, and monitored PNLIP levels in the conditioned media and cell lysates (Fig 2). Consistent with former results, we found that the G233E mutation diminished PNLIP secretion in the HEK 293 variant. Interestingly, HQ also substantially reduced wild-type PNLIP secretion but did not affect intracellular lipase levels.

### Cumulative effect of HQ and misfolded PNLIP on HEK 293AD viability

**Fig 3:**
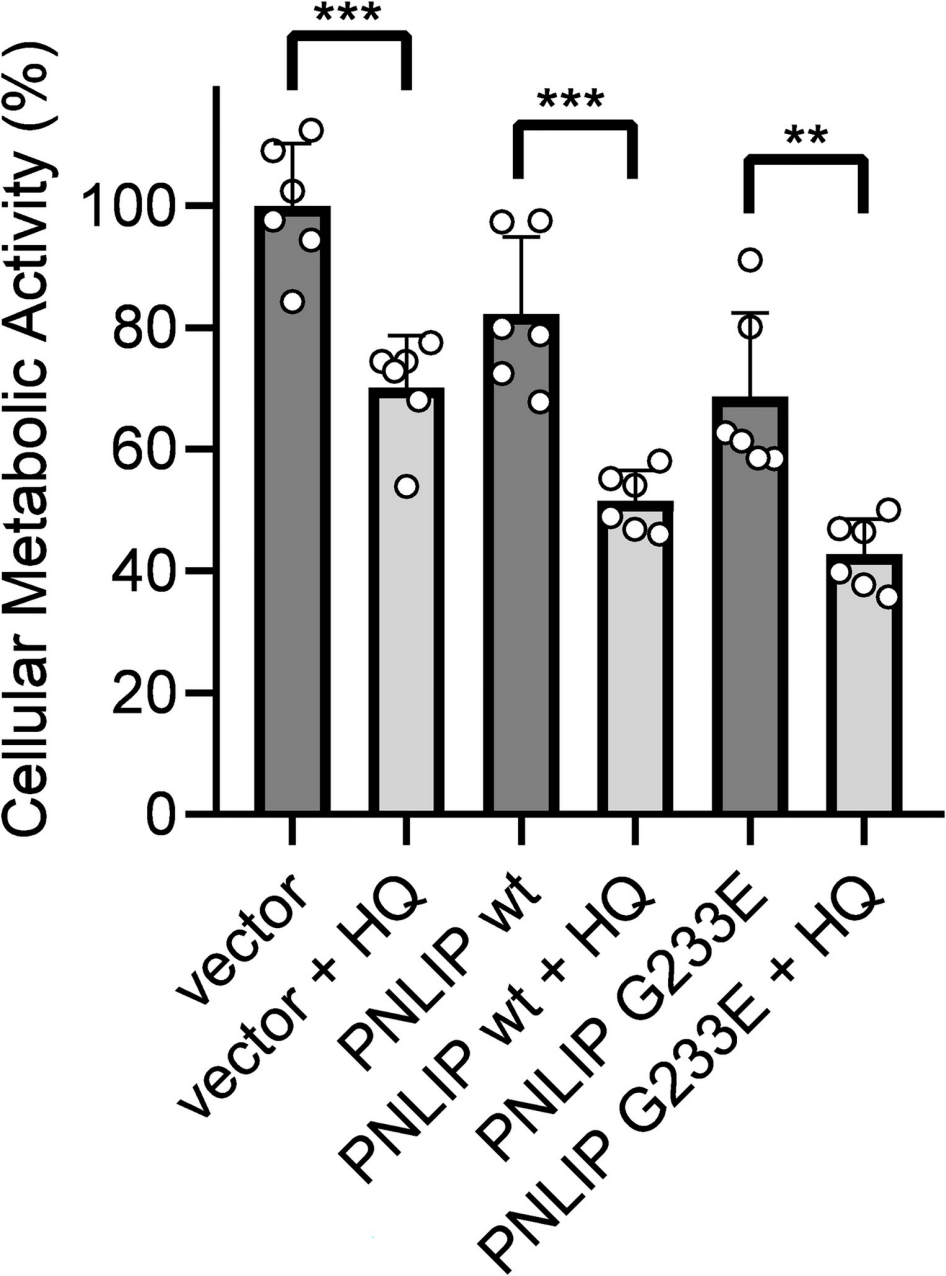
Effect of hydroquinone (HQ) on the viability of wild-type and G233E mutant PNLIP expressing HEK 293AD cells. One day post-transfection, cells were incubated in the absence or presence of 25 μM HQ for 24 h at 37 ˚C, and the levels of cellular metabolic activity were analyzed by MTT assay as described in Materials and Methods. Cells transfected with empty pcDNA3.1(-) vector were used as control. Data points represent the average of 6 independent experiments with SD. **P ≤ 0.01; ***P ≤ 0.001.

In order to study whether the combination of HQ and mutant PNLIP have damaging effect on cell functions, we transiently transfected HEK 293AD cells with wild-type and mutant PNLIP plasmids, and incubated the cells in the absence or presence of 25 μM HQ (Fig 3). At this concentration, HQ has not yet resulted in programmed cell death, thus, providing an opportunity to study early cellular events. Firstly, we monitored HEK 293AD viability by measuring cellular metabolic functions. As a control, we used cells transfected with an empty plasmid DNA. We found that the expression of wild-type PNLIP slightly decreased cellular metabolism by 18%, whereas the expression of mutant PNLIP resulted in a 31% decrease in metabolic activity. Interestingly, the presence of HQ in wild-type and mutant PNLIP expressing cells reduced cell metabolism by 48% and 57%, respectively, whereas HQ alone resulted in only a 30% decrease in metabolic activity.

### Cumulative effect of HQ and misfolded PNLIP on ER stress in HEK 293AD

**Fig 4:**
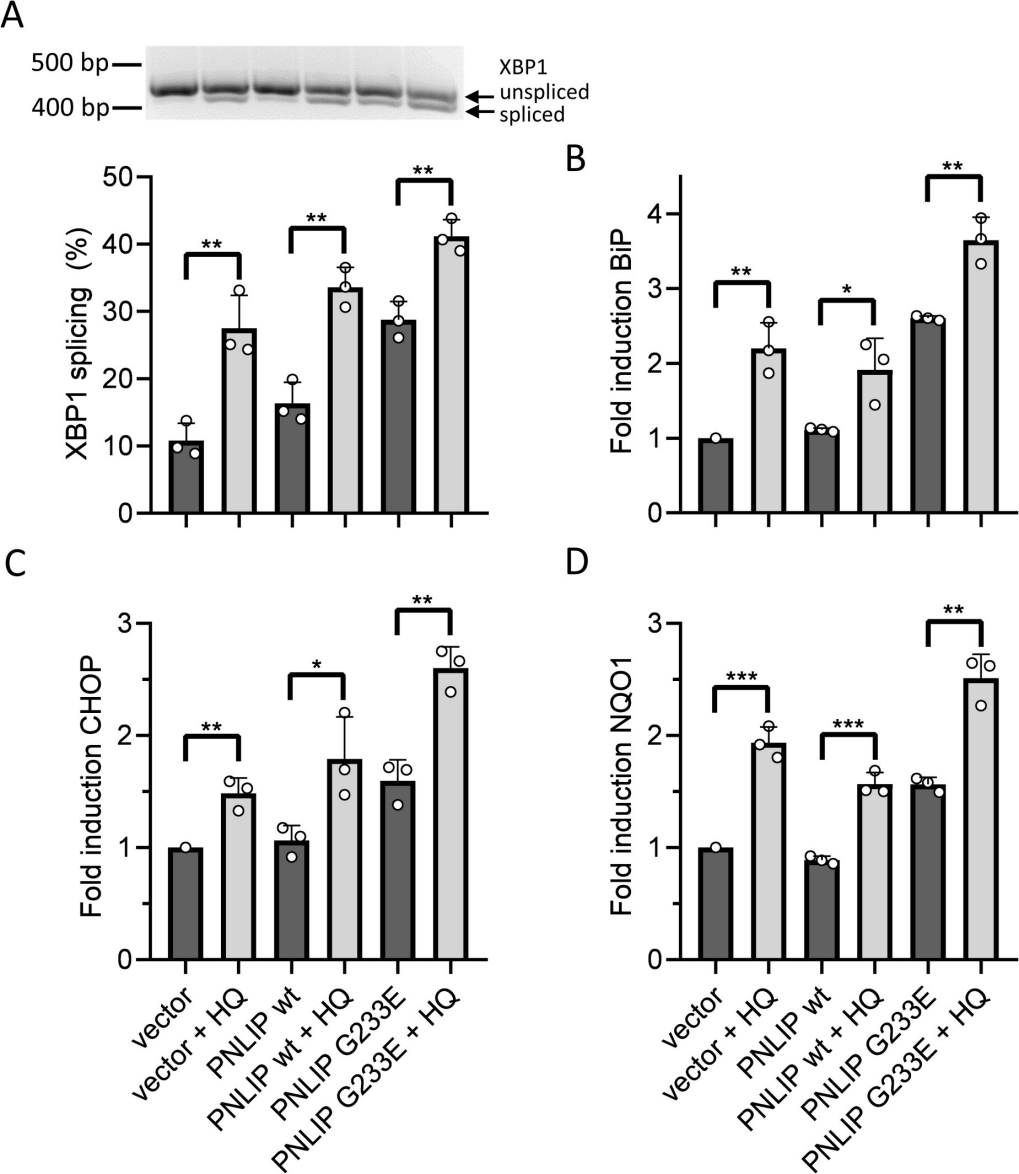
Effect of hydroquinone (HQ) on the levels of ER stress markers in wild-type and G233E mutant PNLIP expressing HEK 293AD cells. One day post-transfection, cells were incubated in the absence or presence of 25 μM HQ for 24 h at 37 ˚C. The mRNA levels of A) XBP1 splicing, B) BiP, C) CHOP and D) NQO1 expressions were assessed as described in Materials and Methods. Cells transfected with empty pcDNA3.1(-) vector were used as control. Data points represent the average of 3 independent experiments with SD. * P ≤ 0.05; **P ≤ 0.01.

Misfolded PNLIP variants, including mutant G233E, resulted in ER stress in HEK 293T and AR42J cells [16]. Consistent with this notion, HQ initiated increased levels of ER stress in several human cell lines [7, 8]. In order to study whether HQ and the expression of G233E mutant PNLIP cooperatively stimulate ER stress, transiently transfected HEK 293AD cells by wild-type and mutant PNLIP plasmids were incubated in the absence or presence of 25 μM HQ, and the levels of ER stress markers XBP1 mRNA splicing and BiP, CHOP and NQO1 expressions were detected (Fig 4 and S1 Table). Cells transfected with the empty expression plasmid served as control. XBP1 mRNA splicing in wild-type PNLIP expressing cells was about 16%, which did not differ substantially from that seen in control cells (Fig 4A). Nevertheless, in mutant PNLIP expressing cells, XBP1 mRNA splicing was increased to 29%. Interestingly, HQ treatment markedly further enhanced XBP1 mRNA splicing in control cells, along with wild-type and mutant PNLIP expressing cells, reaching a maximum yield of 41% in mutant PNLIP expressing cells (Fig 4A and S1 Table).

BiP mRNA levels in wild-type PNLIP expressing HEK 293AD were comparable to control cells transfected with empty plasmid DNA, whereas the expression of mutant PNLIP resulted in a 2.6-fold up-regulation of BiP mRNA levels (Fig 4B). The addition of HQ resulted in 2.2-fold and 1.7-fold induction of BiP mRNA levels in both control and wild-type PNLIP expressing cells, respectively (Fig 4B and S1 Table). In mutant PNLIP expressing cells the addition of HQ led to 1.4-fold increase in BiP expression, which exhibited a 3.6-fold increase in BiP levels compared to control cells. Consistent with previous observations, CHOP mRNA levels in control and wild-type PNLIP expressing cells were comparable, whereas a 1.6-fold increase in CHOP expression was observed in mutant PNLIP expressing cells (Fig 4C). In all cases, HQ treatment resulted in 1.5–1.7-fold increase in CHOP levels (S1 Table). Compared to control cells, the most substantial induction in CHOP expression was observed in cells producing mutant PNLIP in the presence of HQ (Fig 4C). NQO1 mRNA levels in cells transfected by empty and wild-type PNLIP plasmid DNAs were comparable, whereas the mutant PNLIP increased NQO1 expression by 1.6-fold (Fig 4D). The addition of HQ to the cells caused a further up-regulation of NQO1 mRNA (S1 Table). HQ elicited the highest NQO1 expression in cells producing the PNLIP variant (Fig 4D).

### Effect of HQ on the viability and PNLIP secretion of AR42J

**Fig 5:**
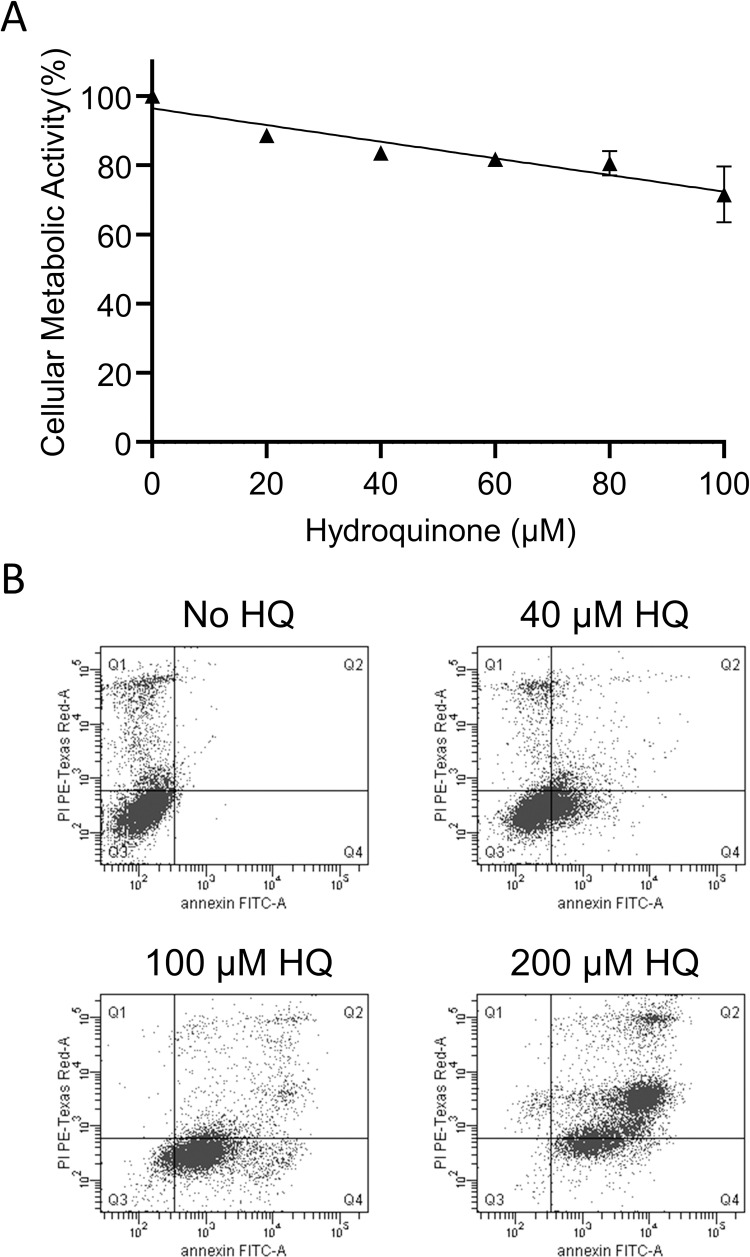
Viability of AR42J cells in the presence of hydroquinone (HQ). Cells were treated with indicated concentrations of HQ for 24 h at 37 ˚C. A) The levels of cellular metabolic activity were analyzed by MTT assay as described in Materials and Methods. Values are expressed as percent of untreated cells. Data points represent the average of three independent experiments with SD. B) Annexin V and propidium iodide stainings of cells were monitored by flow cytometry as described in Materials and Methods. Annexin V and propidium iodide fluorescence are shown on the x and y axes, respectively. Representative images of three experiments are shown.

**Fig 6:**
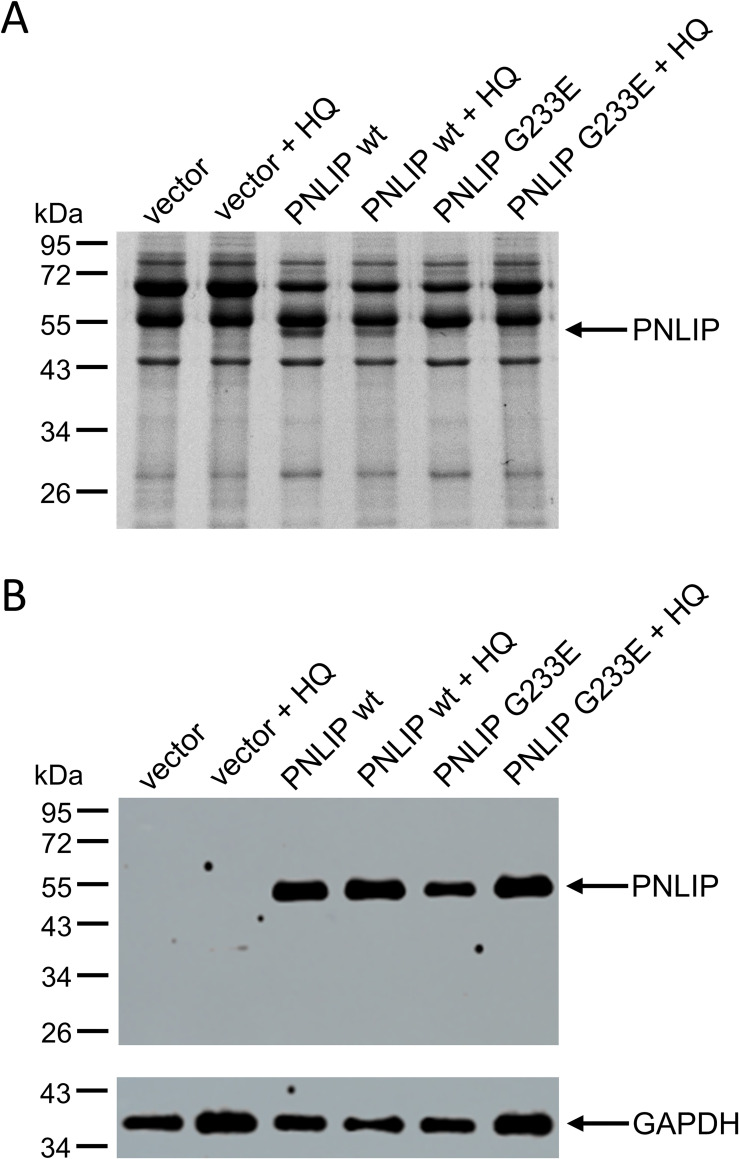
Effect of hydroquinone (HQ) on PNLIP secretion in AR42J cells. One day post-transduction, cells were incubated in the absence and presence of 40 μM HQ for 24 h at 37 ˚C. A) PNLIP protein in the conditioned media was analyzed by SDS-PAGE and Coomassie staining as described in Materials and Methods. B) PNLIP and GAPDH proteins in cell lysates were monitored by SDS-PAGE and immunoblotting as described previously [16].

AR42J rat pancreatic cells, differentiated by dexamethasone, were also used to study the cellular effects of HQ. We incubated AR42J in the absence or presence of increasing concentrations of HQ, and monitored cell viability by measuring their metabolic activity (Fig 5A). By increasing HQ concentration to 100 μM, only a slight decrease in cell metabolism was observed, which suggests that AR42J cells are more resilient to the stressor than HEK 293AD cells. In order to study whether programmed cell death of AR42J also occurs in the presence of increasing concentrations of HQ, the extents of FITC annexin V and propidium iodide staining of the cells were evaluated by flow cytometry (Fig 5B). Up to 40 μM HQ, most of the cells remained unstained by the reagents. The majority of the cells first became annexin V positive in the presence of 100 μM HQ, and also reacted with propidium iodide at an even higher HQ concentration. These results indicated that HQ initiates programmed cell death in AR42J at much higher concentrations than observed in HEK 293AD cells. To study the effect of HQ on the expression and secretion of PNLIP, we incubated AR42J cells expressing wild-type and mutant PNLIP with 40 μM HQ and monitored PNLIP levels in the conditioned media and cell lysates (Fig 6). In agreement with our previous observation [16], the G233E misfolding mutation inhibited PNLIP secretion in AR42J cells. In addition, HQ treatment markedly reduced PNLIP secretion, whereas the intracellular lipase levels remained unaffected.

### Cumulative effect of HQ and G233E PNLIP variant on AR42J viability

**Fig 7:**
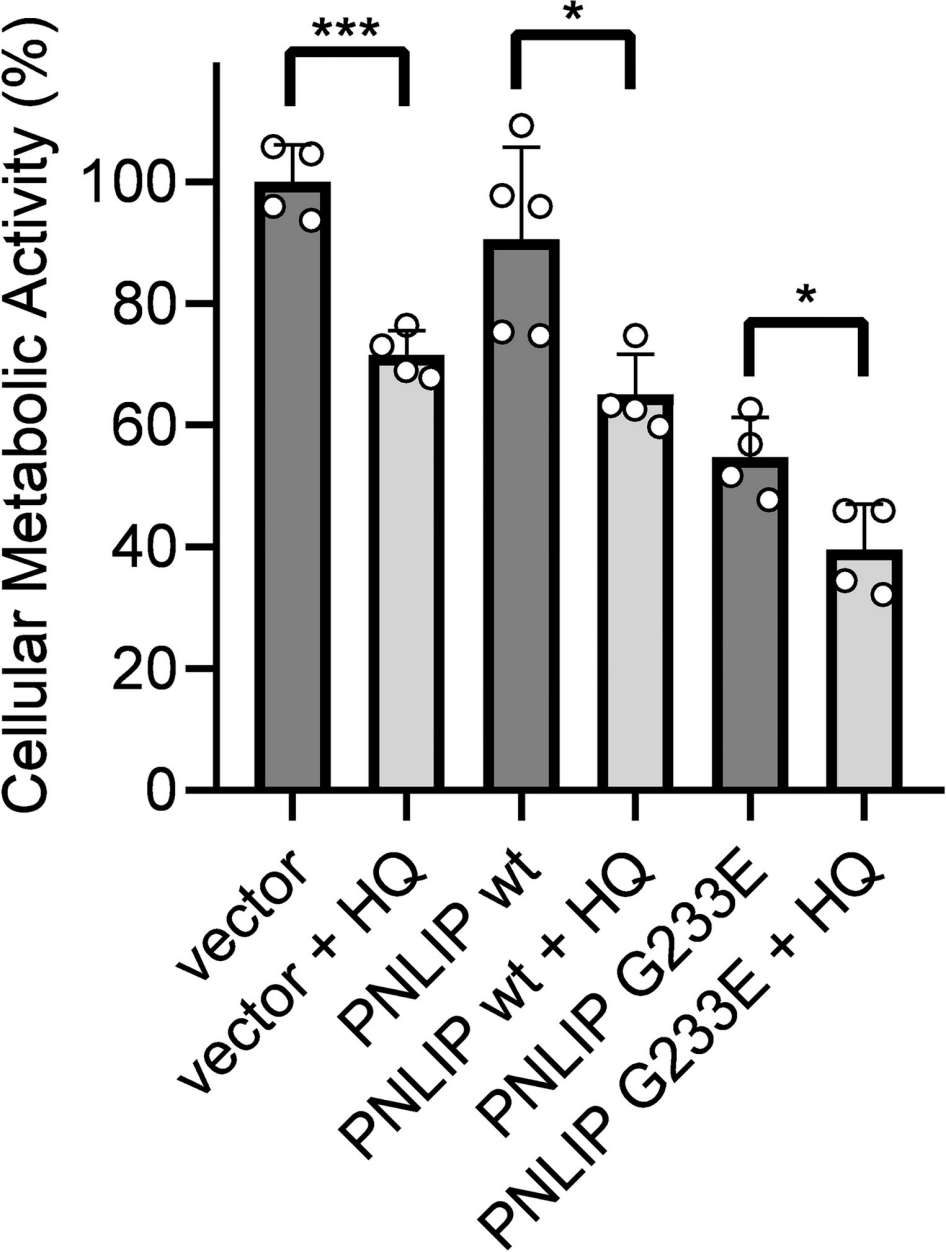
Effect of hydroquinone (HQ) on the viability of wild-type and G233E mutant PNLIP expressing AR42J cells. One day post-transduction, cells were incubated in the absence or presence of 40 μM HQ for 24 h at 37 ˚C, and the levels of cellular metabolic activity were analyzed by MTT assay as described in Materials and Methods. Cells transfected with empty adenovirus vector were used as control. Data points represent the average of four independent experiments with SD. * P ≤ 0.05; ***P ≤ 0.001.

In order to study if HQ and the expression of G233E mutant PNLIP have a cooperative effect on AR42J viability, the cells were transduced with wild-type and mutant PNLIP-adenoviruses, and then incubated in the absence or presence of 40 μM HQ (Fig 7). As control, cells transduced by empty adenovirus were used. The addition of HQ decreased AR42J viability by reducing metabolic functions by 28%. The expression of the wild-type PNLIP caused only a slight and insignificant decrease in cell metabolism, whereas the expression of the mutant PNLIP reduced AR42J metabolic activity by 45%. The addition of HQ to the wild-type and mutant PNLIP expressing cells further decreased cell viability. The most prominent loss of viability was observed in cells expressing the mutant PNLIP, in which HQ reduced metabolic activity by 60%.

### Cumulative effect of HQ and misfolded PNLIP on ER stress in AR42J cells

**Fig 8:**
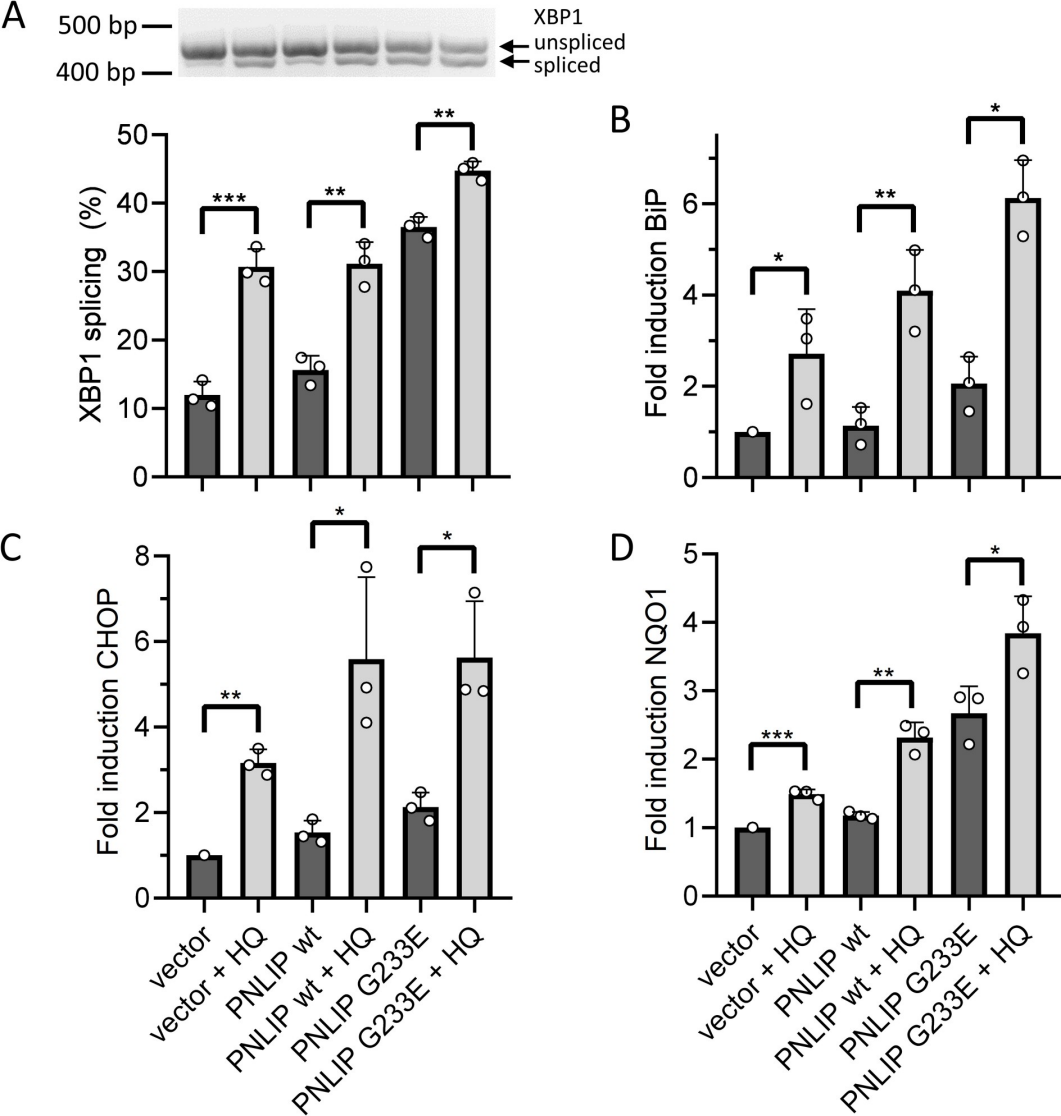
Effect of hydroquinone (HQ) on the levels of ER stress markers in wild-type and G233E mutant PNLIP expressing AR42J cells. One day post-transduction, cells were incubated in the absence or presence of 40 μM HQ for 24 h at 37 ˚C. The mRNA levels of A) XBP1 splicing, B) BiP, C) CHOP and D) NQO1 expressions were assessed as described in Materials and Methods. Cells infected with empty adenovirus vector were used as control. Data points represent the average of three independent experiments with SD. * P ≤ 0.05; **P ≤ 0.01; ***P ≤ 0.001.

To confirm the cooperative effect of G233E PNLIP variant and HQ on AR42J ER stress levels, we determined the intracellular mRNA levels of XBP1 splicing, BiP, CHOP and NQO1 (Fig 8 and S2 Table). Cells transduced by empty adenoviruses served as controls. The extent of XBP1 mRNA splicing was about 12–16% in both control and wild-type PNLIP expressing cells, and increased to 37% in mutant lipase producing cells. The presence of HQ increased XBP1 mRNA splicing to 31% in both control and wild-type PNLIP producing cells, whereas HQ escalated XBP1 splicing to 45% in mutant PNLIP expressing cells (Fig 8A). BiP mRNA expression in wild-type PNLIP expressing cells was comparable to that in control cells (Fig 8B). In contrast, a 2.1-fold increase in BiP expression was observed in mutant PNLIP expressing cells. HQ significantly induced BiP expression by 2.7–3.6-fold in all cases (Fig 8B and S2 Table). The highest level of BiP mRNA was detected in cells expressing the mutant PNLIP, where the addition of HQ 6.1-fold upregulated BiP mRNA levels compared to control cells. The mRNA levels of CHOP in cells expressing wild-type and mutant PNLIP were slightly induced by 1.5–2.1-fold, compared to control cells (Fig 8C). The addition of HQ substantially further increased CHOP expression in both wild-type and mutant PNLIP expressing cells (Fig 8C and S2 Table). In agreement with previous findings, NQO1 mRNA levels increased 2.7-fold in mutant PNLIP expressing cells, while remaining unchanged in wild-type PNLIP expressing cells (Fig 8D). HQ substantially further induced NQO1 mRNA levels in cells expressing wild-type and mutant PNLIP (Fig 8D and S2 Table). The highest NQO1 mRNA levels (3.8-fold) were detected in HQ-treated mutant PNLIP expressing cells.

## Discussion

Cigarette smoke is among the most common causes of chronic pancreatitis. Several studies indicated that smoking reduces pancreatic fluid secretion, stimulates fibrosis and induces the expression of inflammatory cytokines in the pancreas [20, 21]. A recent publication also reported that smoking accelerates the development of pancreatic exocrine insufficiency in chronic pancreatitis [22]. Nicotine and nicotine-derived nitrosamine ketone are the most studied cigarette smoke constituents, which predispose to intracellular retention and premature activation of secretory proteases in pancreatic acinar cells, thereby increasing the risk for pancreatitis [23]. The detrimental effect of other smoke constituents on the pancreas is less known. Acrolein, crotonaldehyde and HQ are among the most cytotoxic chemicals in cigarette smoke [5]. Smoke from cigarettes is estimated to contain about 18–98 μg acrolein, 1–53 μg crotonaldehyde and 110–300 μg HQ per cigarette [24–26]. Lugea *et al*. demonstrated that acrolein upregulates the pro-apoptotic ER stress marker CHOP and induces pancreatic cell death [4]. In addition, another publication reported that HQ diminishes the antioxidant defense system, and activates apoptotic caspases in the pancreas [27]. Following low-dose oral administration, plasma HQ concentrations of 60–70 μM are readily reached in rodents [28]. Although, smoking results in somewhat lower transient rises of HQ concentrations in the blood plasma, it supplements HQ exposures from dietary sources [25]. HQ is a cytotoxic chemical, which can be converted to semiquinone radicals in the cells causing oxidative stress and cell damage [29]. HQ can result in chromosomal aberrations such as mitotic recombination, DNA breaks and chromosomal translocations [30]. In addition, HQ may inhibit the activity of proteasome, and induce ER stress and the formation of autophagosomes [7].

Although mammalian cell lines are less relevant for modeling molecular pathways than primary cells, those are frequently used to study the transient expression and secretion of pancreatic secretory proteins. In our present study, first we monitored the viability of HEK 293AD cells; a common variant of the parental cell line, in the presence of increasing concentrations of acrolein, crotonaldehyde and HQ. Our results demonstrated that all three stressors reduced cell viability in a dose-dependent manner. Crotonaldehyde and HQ had the most detrimental effects. HEK 293AD cells were less viable than AR42J cells in the presence of HQ. It has already been shown that some cell types are less tolerant to hydroquinone due to the dysfunction of DNA repair pathways [30]. Due to its favorable shelf life, only the cellular effects of HQ were investigated in further experiments. Our observations showed that HQ treatment diminished PNLIP secretion in transfected HEK 293AD and AR42J pancreatic cells without affecting lipase expression. We hypothesized that reduced cell viability is caused by ER stress, therefore, we monitored the levels of ER stress markers of the unfolded protein response pathways in both cell lines. We found that HQ induces ER stress in both cell lines, demonstrated by elevated levels of XBP1 mRNA splicing, and increased expressions of BiP, CHOP and NQO1. Consistent with this notion, HQ reduced cell viability by inducing programmed cell death, shown by enhanced FITC annexin V staining and subsequent propidium iodide staining of the cells.

Missense mutations in pancreatic secretory enzymes such as CPA1, PRSS1, CEL and PNLIP may lead to protein misfolding and the development of ER stress, which may predispose to chronic pancreatitis [9]. Accumulating evidence showed that the role of these susceptibility factors in initiation of the disease may vary depending on their expression. CPA1 and PRSS1 are highly expressed by pancreatic acinar cells [31]. Consequently, heterozygous CPA1 and PRSS1 misfolding mutations are considered as strong risk factors, and are solely capable of inducing chronic pancreatitis. CEL and PNLIP are expressed to a lesser extent by the exocrine pancreas [31, 32]. Genetic analyses suggested that heterozygous CEL misfolding mutations may predispose to the disease, but do not impel pancreatitis *per se* [33]. Recently, we identified a subset of rare heterozygous PNLIP mutations, in both chronic pancreatitis patients and healthy controls with reduced lipase secretion [15]. Further functional characterizations revealed that poor lipase secretion was due to PNLIP misfolding and intracellular accumulation as insoluble protein aggregates [16]. The mutations induced ER stress indicated by increased levels of XBP1 mRNA splicing, and BiP mRNA expression. Although ER stress in pancreatic acinar cells predispose to chronic pancreatitis, the mixed clinical phenotype of PNLIP variants, together with moderate lipase expression, suggested that these heterozygous mutations are unlikely to induce pancreatitis alone.

We hypothesized that smoke constituent HQ and the misfolding genetic susceptibility cooperatively increase the propensity to chronic pancreatitis. We expressed the wild-type and misfolding G233E PNLIP variant in HEK 293AD and AR42J cells in the absence and presence of HQ, and monitored cell viability and the levels of ER stress. In cells expressing the mutant PNLIP, cell viability decreased more notably than in cells expressing the wild-type PNLIP. HQ further reduced cell viability, which was most substantial in cells expressing the mutant PNLIP. Expression of the mutant PNLIP increased the levels of ER stress markers XBP1 mRNA splicing and the expression of BiP, CHOP and NQO1 in both cell lines. HQ further enhanced the extent of ER stress, reaching the highest levels in mutant PNLIP expressing cells. These observations demonstrated the cumulative detrimental effect of HQ and misfolding PNLIP variant.

Chymotrypsin C (CTRC) is a minor chymotrypsin isoform secreted by the exocrine pancreas. CTRC prevents premature intrapancreatic protease activation and chronic pancreatitis by degrading trypsinogen [34]. Loss-of-function mutations in CTRC predispose to the disease [35]. Recently, LaRusch *et al*. demonstrated that chronic pancreatitis more likely develops in smokers carrying the common pathogenic c.180C>T CTRC mutation than in non-smoker carriers [36]. Additionally, non-allelic homozygous recombination of CEL and its neighboring pseudogene CELP results in a hybrid protein CEL-HYB1 characterized by impaired activity, secretion defect, misfolding, and increased susceptibility for chronic pancreatitis [37]. Carriers of CEL-HYB1 do not always develop chronic pancreatitis suggesting that the presence of other risk factors are required for disease development. In agreement with the previous observation, Tjora *et al*. reported a CEL-HYB1 heterozygous misfolding mutation carrier with recurrent abdominal pain whose symptom disappeared on quitting smoking [33]. Lugea *et al*. described that cigarette smoke promotes cell death of ethanol sensitized pancreatic acinar cells, via the activation of ER stress pathways [4]. Consistent with this notion, Orekhova *et al*. demonstrated that alcohol feeding of mice expressing the misfolding N256K human CPA1 variant revealed more severe pancreatitis, characterized by even higher levels of ER stress than CPA1 N256K mice on a control liquid diet [38].

Taken together, our results demonstrated that genetic susceptibility may sensitize pancreatic acinar cells to injury, and in the presence of cigarette smoke constituents, it promotes a more severe ER stress, which may accelerate to chronic pancreatitis.

## Supporting information

S1 Table Effect of hydroquinone (HQ) on XBP1 mRNA splicing and BiP, CHOP and NQO1 expressions in HEK 293AD cells. Values are expressed as foldchanges normalized to transfected cells without HQ.
(DOCX)

S2 Table Effect of hydroquinone (HQ) on XBP1 mRNA splicing and BiP, CHOP and NQO1 expressions in AR42J cells. Values are expressed as foldchanges normalized to transduced cells without HQ.
(DOCX)

S1 Dataset (XLSX)

S1 File (PDF)
