# Supplementary material for: Cigarette smoke toxin hydroquinone and misfolding pancreatic lipase variant cooperatively promote endoplasmic reticulum stress and cell death
Source: PLoS One. 2022 Jun 15;17(6):e0269936. doi: 10.1371/journal.pone.0269936 (PMC9200355; doi:10.1371/journal.pone.0269936)
Supplement: S1 Table — Values are expressed as foldchanges normalized to transfected cells without HQ. (DOCX) [file pone.0269936.s001.docx]

| HEK 293AD | XBP1 splicing | BiP foldchange | CHOP foldchange | NQO1 foldchange |
| --- | --- | --- | --- | --- |
| Vector | 1.0 | 1.0 | 1.0 | 1.0 |
| Vector + HQ | **2.6** | **2.2** | **1.5** | **1.9** |
| PNLIP wt | 1.0 | 1.0 | 1.0 | 1.0 |
| PNLIP wt + HQ | **2.1** | **1.7** | **1.7** | **1.8** |
| PNLIP G233E | 1.0 | 1.0 | 1.0 | 1.0 |
| PNLIP G233E + HQ | **1.4** | **1.4** | **1.6** | **1.6** |

**S1 Table.** Effect of hydroquinone (HQ) on XBP1 mRNA splicing and BiP, CHOP and NQO1 expressions in HEK 293AD cells. Values are expressed as foldchanges normalized to transfected cells without HQ.
