# Supplementary material for: Cigarette smoke toxin hydroquinone and misfolding pancreatic lipase variant cooperatively promote endoplasmic reticulum stress and cell death
Source: PLoS One. 2022 Jun 15;17(6):e0269936. doi: 10.1371/journal.pone.0269936 (PMC9200355; doi:10.1371/journal.pone.0269936)
Supplement: S2 Table — Values are expressed as foldchanges normalized to transduced cells without HQ. (DOCX) [file pone.0269936.s002.docx]

**S2 Table.** Effect of hydroquinone (HQ) on XBP1 mRNA splicing and BiP, CHOP and NQO1 expressions in AR42J cells. Values are expressed as foldchanges normalized to transduced cells without HQ.

| AR42J | XBP1 splicing | BiP foldchange | CHOP foldchange | NQO1 foldchange |
| --- | --- | --- | --- | --- |
| Vector | 1.0 | 1.0 | 1.0 | 1.0 |
| Vector + HQ | **2.6** | **2.7** | **3.2** | **1.5** |
| PNLIP wt | 1.0 | 1.0 | 1.0 | 1.0 |
| PNLIP wt + HQ | **2.0** | **3.6** | **3.6** | **2.0** |
| PNLIP G233E | 1.0 | 1.0 | 1.0 | 1.0 |
| PNLIP G233E + HQ | **1.2** | **3.0** | **2.6** | **1.4** |
