# Supplementary material for: Cigarette smoke toxin hydroquinone and misfolding pancreatic lipase variant cooperatively promote endoplasmic reticulum stress and cell death
Source: PLoS One. 2022 Jun 15;17(6):e0269936. doi: 10.1371/journal.pone.0269936 (PMC9200355; doi:10.1371/journal.pone.0269936)
Supplement: S1 File — (PDF) [file pone.0269936.s004.pdf]

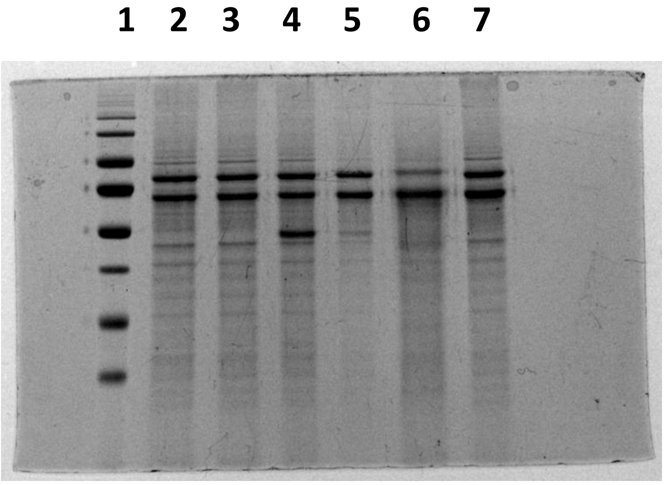

<u>Loading order</u>

1. **Molecular weight standard** (PageRuler #26616)
2. **Vector**
3. **Vector + HQ**
4. **Wild type PNLIP**
5. **Wild type PNLIP + HQ**
6. **PNLIP G233E**
7. **PNLIP G233E + HQ**

Fig1A

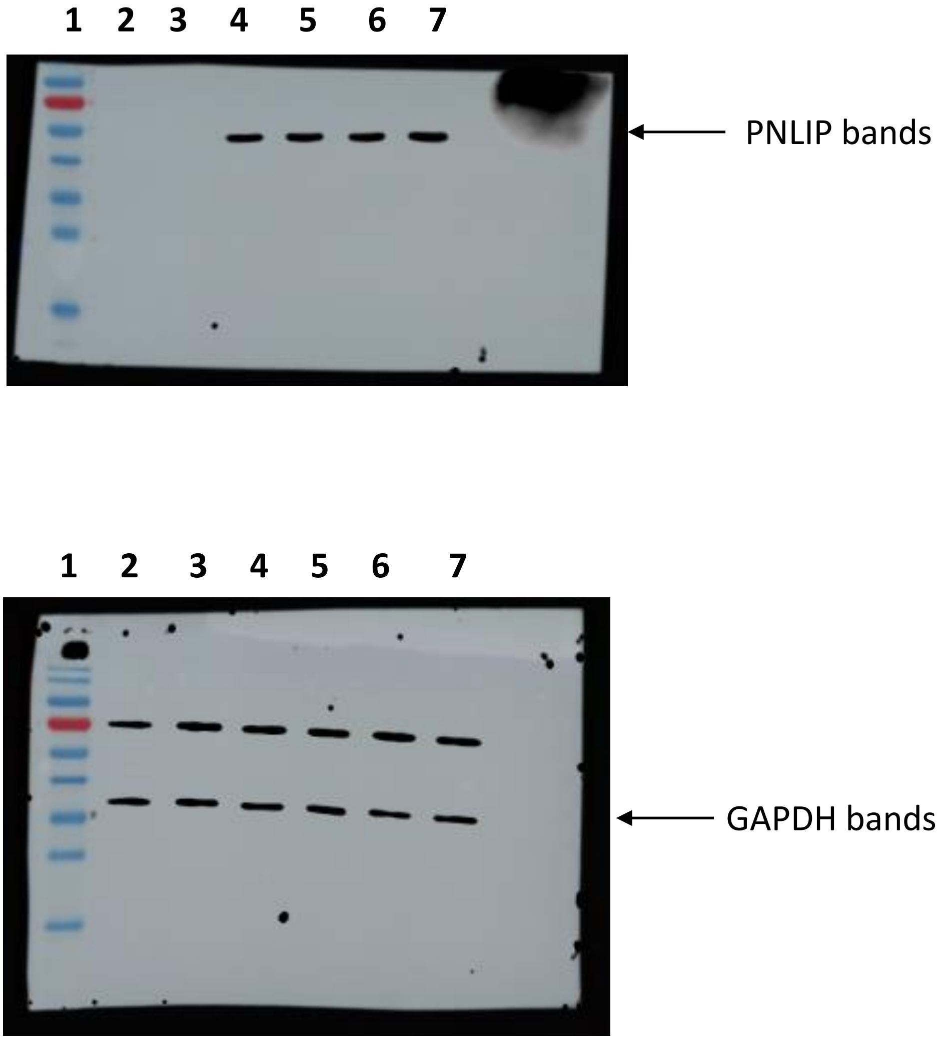

Loading order

1. **Molecular weight standard** (PageRuler #26616)
2. **Vector**
3. **Vector + HQ**
4. **Wild type PNLIP**
5. **Wild type PNLIP + HQ**
6. **PNLIP G233E**
7. **PNLIP G233E + HQ**

Fig2B

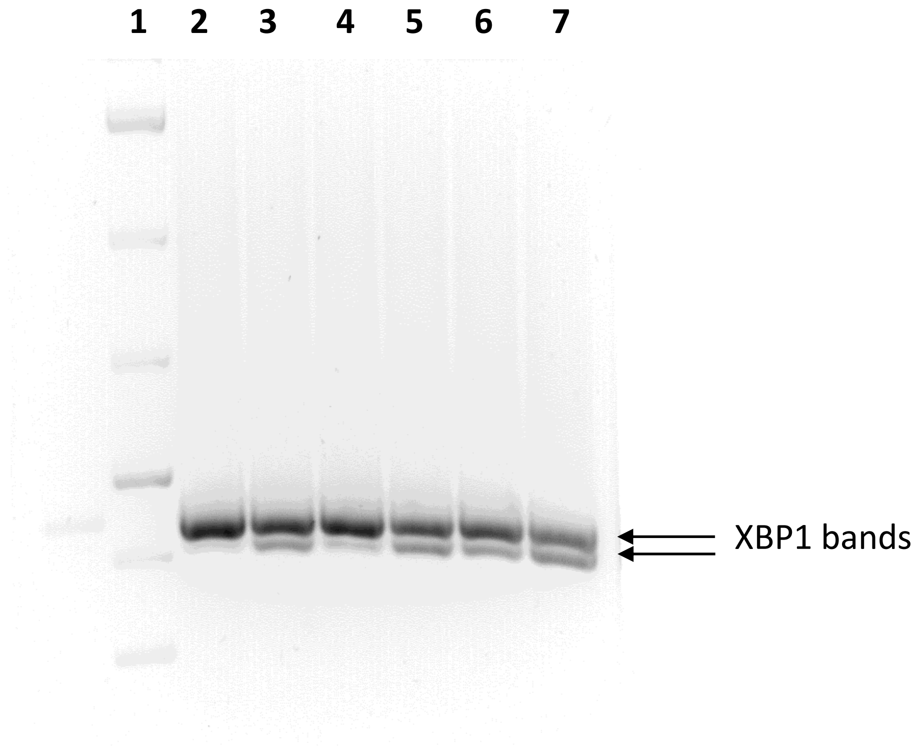

<u>Loading order</u>

1. **DNA Ladder** (GeneRuler 1kbp+ #SM1331)
2. **Vector**
3. **Vector + HQ**
4. **Wild type PNLIP**
5. **Wild type PNLIP + HQ**
6. **PNLIP G233E**
7. **PNLIP G233E + HQ**

Fig4A

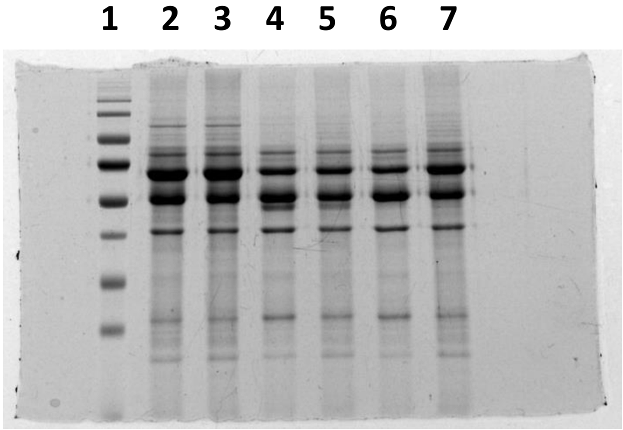

Loading order

1. **Molecular weight standard** (PageRuler #26616)
2. **Vector**
3. **Vector + HQ**
4. **Wild type PNLIP**
5. **Wild type PNLIP + HQ**
6. **PNLIP G233E**
7. **PNLIP G233E + HQ**

Fig6A

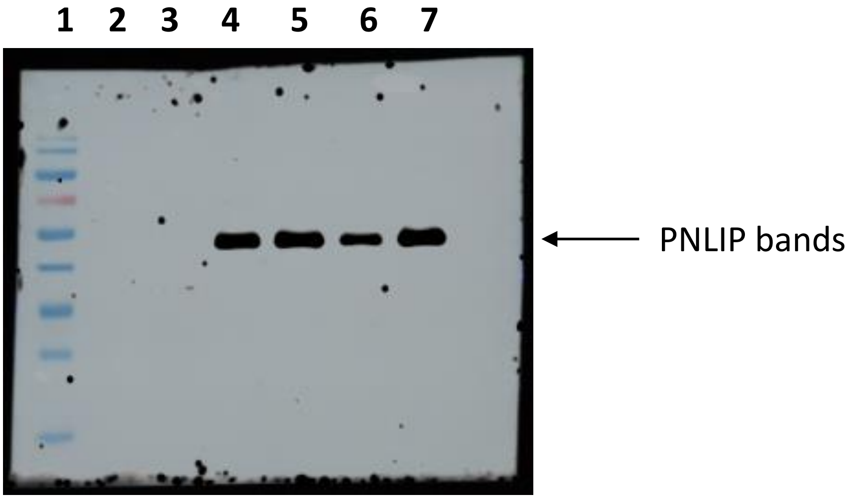

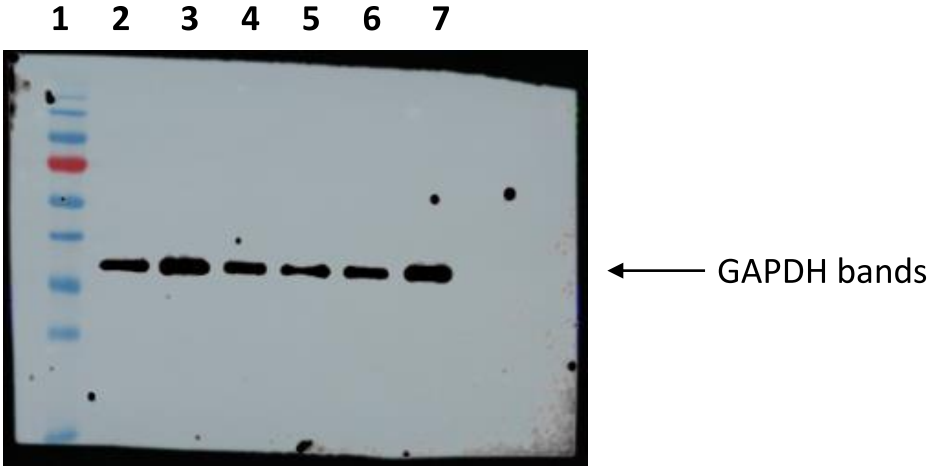

<u>Loading order</u>

1. **Molecular weight standard** (PageRuler #26616)
2. **Vector**
3. **Vector + HQ**
4. **Wild type PNLIP**
5. **Wild type PNLIP + HQ**
6. **PNLIP G233E**
7. **PNLIP G233E + HQ**

Fig6B

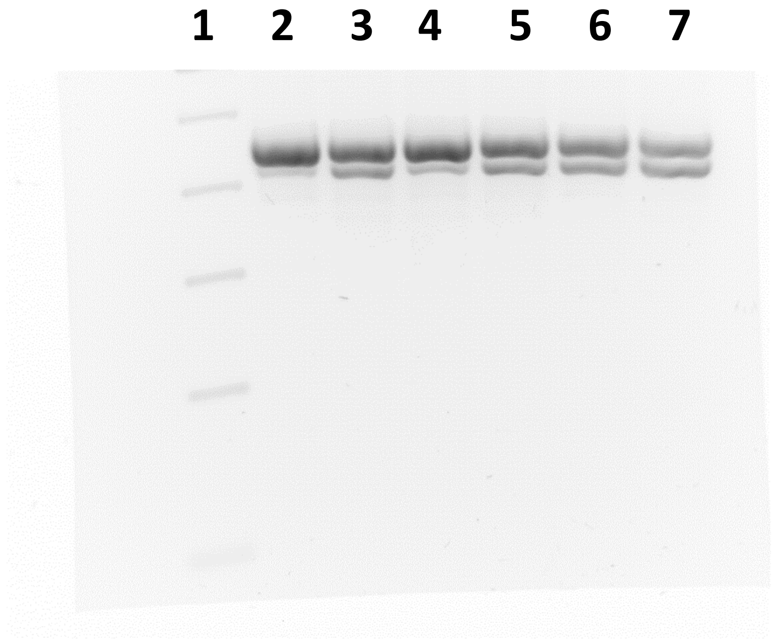

Loading order

1. **DNA Ladder** (GeneRuler 1kbp+ #SM1331)
2. **Vector**
3. **Vector + HQ**
4. **Wild type PNLIP**
5. **Wild type PNLIP + HQ**
6. **PNLIP G233E**
7. **PNLIP G233E + HQ**

Fig8A
